# A Comprehensive Review with Future Insights on the Processing and Safety of Fermented Fish and the Associated Changes

**DOI:** 10.3390/foods12030558

**Published:** 2023-01-27

**Authors:** Sharon Xi Ying Chan, Nursyah Fitri, Nurul Syahidah Mio Asni, Nor Hafiza Sayuti, Ummi Kalthum Azlan, Wasim S. M. Qadi, Esraa Adnan Dawoud Dawoud, Nurkhalida Kamal, Murni Nazira Sarian, Mohd Aizuddin Mohd Lazaldin, Chen Fei Low, Sarahani Harun, Hamizah Shahirah Hamezah, Emelda Rosseleena Rohani, Ahmed Mediani

**Affiliations:** 1 Department of Biosciences, Faculty of Science, Universiti Teknologi Malaysia, Skudai 81310, Malaysia; 2 Institute of Systems Biology (INBIOSIS), Universiti Kebangsaan Malaysia (UKM), Bangi 43600, Malaysia; 3 Department of Food Science, Faculty of Science and Technology, Universiti Kebangsaan Malaysia, Bangi 43650, Malaysia; 4 Faculty of Pharmacy, Universiti Kebangsaan Malaysia, Bangi 43650, Malaysia

**Keywords:** fish, fermentation, health benefits, omics, processing, preservation, sensory attributes, safety

## Abstract

As an easily spoiled source of valuable proteins and lipids, fish is preserved by fermentation in many cultures. Over time, diverse types of products have been produced from fish fermentation aside from whole fish, such as fermented fish paste and sauces. The consumption of fermented fish products has been shown to improve both physical and mental health due to the composition of the products. Fermented fish products can be dried prior to the fermentation process and include various additives to enhance the flavours and aid in fermentation. At the same time, the fermentation process and its conditions play a major role in determining the quality and safety of the product as the compositions change biochemically throughout fermentation. Additionally, the necessity of certain microorganisms and challenges in avoiding harmful microbes are reviewed to further optimise fermentation conditions in the future. Although several advanced technologies have emerged to produce better quality products and easier processes, the diversity of processes, ingredients, and products of fermented fish warrants further study, especially for the sake of the consumers’ health and safety. In this review, the nutritional, microbial, and sensory characteristics of fermented fish are explored to better understand the health benefits along with the safety challenges introduced by fermented fish products. An exploratory approach of the published literature was conducted to achieve the purpose of this review using numerous books and online databases, including Google Scholar, Web of Science, Scopus, ScienceDirect, and PubMed Central, with the goal of obtaining, compiling, and reconstructing information on a variety of fundamental aspects of fish fermentation. This review explores significant information from all available library databases from 1950 to 2022. This review can assist food industries involved in fermented fish commercialization to efficiently ferment and produce better quality products by easing the fermentation process without risking the health and safety of consumers.

## 1. Introduction

Fish and fish products are regularly consumed foods as a reliable source of nutrients. They are good sources of polyunsaturated fatty acids such as omega-3, proteins, vitamin D, and selenium, which can contribute to a healthy diet [1,2]. However, fish degrades and perishes in a short amount of time. Furthermore, global fish production has increased from 20 million tons in 1950, to 171 million tons in 2016. Thus, fish consumption has also increased globally, whereby each person consumed up to 20.2 kg per person in 2015, compared to 9 kg per person in 1961 [3]. Even so, higher fish production produces a higher cost of fish waste. Hence, preserving fish is becoming more important than before to utilise this nutrient source, which otherwise may end up rotting in the landfill. For this purpose, fermenting the fish can be a solution to stretch the short shelf life of fresh fish. This involves fermentation, which is a metabolic process that acquires energy from organic compounds without the use of any external oxidizing agent [4]. It is a processing method for various crops and food products that has been utilised since ancient times. Fish fermentation itself is an ancient technology, with evidence of the widespread use of fish fermentation during Japan’s Yayoi period, from 300 BCE to 300 CE [5]. Thus, fermented fish has long been a staple diet for some parts of the world. The fermented fish consumed can be in a variety of shapes and sizes, and most importantly, use diverse methods of preparation and fermentation. Even the end products can be of different consistency, which include fish sauce, paste, and solid dried fermented fish, such as katsuobushi, from Japan [6]. Fermentation can be a better preservation method for fish than freezing, smoking, and drying, as it is able to preserve the fish by enhancing the nutritional value through beneficial microorganisms involved in fermentation [2,7,8].

Fermentation of fish is advantageous, as it can prolong the shelf life of fish and reduce fish waste. Furthermore, fermented fish products can contribute to the economy of the country of origin. As an example, in the fermented fish sauce industry, Thailand’s fish sauce, or nampla, has been popular in most Western countries, especially the United States. This makes Thailand one of the leaders in the fish sauce industry in the world, which contributes to the Thai economy [9]. Additionally, fermenting fish contributes to changes in sensory attributes of the fish, which can be desirable and preferable, based on the types of fermented fish. The fish flavours can be enhanced by the by-products of the fermentation by microorganisms. The degradation of nutrients such as protein and lipid in fish contributes to the production of some organoleptic compounds, thus causing changes in its sensory properties [10]. These changes are, more importantly, measurable parameters for the assessment of quality attributes of the fermented fish. Certain changes can be favourable, and others are undesirable, such as the production of off-flavours and rancid odour; and, therefore, should be prevented by altering the fermentation parameters [10]. As an example, dimethyl trisulfide, 2,3-butanedione, and 2-methylpropanal are examples of organoleptic volatile compounds that have been detected in samples of fermented fish miso and nampla. The compound is responsible for the fishy odour, caramel and nutty flavour in the fish miso and nampla, respectively [11]. Thus, fish fermentation can provide an array of opportunities to the fishery industry and local businesses. This rich source of nutrients can be fermented to stretch the shelf-life of fish and add value to its nutritional benefits and flavour. Therefore, exploring the variety of fermented fish products and changes occurring after fermentation are the main purposes of this review, to enlighten the process of fermentation itself. This review also covers the safety and challenges of fermented fish and fish products.

## 2. Fermented Fish and Fish Products

Fermented fish is a delicacy for people all over the world, especially in Asian and African countries, but also in Northern Europe. Notably, distinctive features distinguish it from other fish products preserved differently, such as dried fish. These characteristics include the appearance of the final product, packaging methods, and complex flavours of the products. Fermented fish products usually come in three different forms in terms of appearances or texture (Table 1). These include solid (whole), in which the structure of the fish is mostly retained; as well as other forms, such as fish paste and fish sauce [5,12,13]. Meanwhile, dried fish and frozen fish are mostly like the fresh form [14,15].

Furthermore, different fermentation process parameters determine the appearance and quality of the final products. The fermentation parameters vary among different types of fermented fish products, which contributes to the distinctive sensory characteristics. The fermentation of fish is generally categorised into natural fermentation and controlled fermentation, where the former is traditional, and the latter is highly commercialised. The fermentation parameters include the salt ratio, temperature, starter culture, and length of fermentation period [5]. These are different in the case of dried fish and frozen fish, where the fermentation process does not occur [16]. Consequently, the final appearance of the fermented fish products affects its packaging. Fish sauce and fish paste are stored in bottles and jars, whereas solid fermented fish can be sealed in cans [17,18]. However, most dried fish in the market are sold in the open-air markets without proper packaging of the product [19]. Frozen fish needs a freezing temperature below 0 °C as its storage condition [16]. Therefore, fermented fish products can be differentiated and stand out from other preserved fish products in terms of its physical properties; because it is fermented, it has better packaging and flavour than other preserved fish products.

**Table 1:** Summary of various fermented fish products and their fermentation parameters.

Products	Origin	Types of Products	Types of Fish	Pre-Treatment/ Additive	Fermentation Time	Temperature	Ref.
Nampla	Thailand	Fish sauce	*Stolephrous* spp. *Ristrelliger* spp. *Cirrhinus* spp.	Salt or brine throughout the process	6–18 months, depending on the size of fish	Ambient temperature	[9,20]
Budu	Malaysia	Fish sauce/paste	*Stolephorus* spp. *Sardinella* spp. *Decaterus macrosoma*	Fish added with salt before fermentation, and added with palm sugar, tamarind, and monosodium glutamate as flavourings	6–12 months	30–40 °C	[21,22]
Bakasang	Indonesia	Fish sauce/paste	*Katsuwonus pelamis* L.	Preconditioned in a warm place	7 days	30–50 °C	[23,24]
Yu-Lu	China	Fish sauce	*Engraulis japonicus* *Channa asiatica*	Preconditioned with salt at a 1:3 salt-to-fish ratio	2–6 months	20–25 °C	[25,26]
Shidal	India	Whole fish	*Puntius* spp. *Setipinna phasa*	Air-tight earthen container	3–5 months	Room temperature	[27,28]
Jeotgal (e.g., myeolchi-jeot)	Korean	Whole fish	Anchovies	Only salt, or with the addition of Korean red peppers, soy sauce, and/or malted rice	Two months for jeotgal with low-salt levels (6–18%), and a few years for jeotgal with a high-salt content (over 20%)	10–30 °C	[29,30]
Katsuobushi	Japan	Whole fish	*Euthynnus pelamis* *Katsuwonus pelamis* *Euphonia affinis* *Auxis rochei* *Auxis thazard* *Sarda orientalis*	Fish are dried (Arabushi)	6 months	Ambient temperature	[6,31]
Feseekh	Egypt	Whole fish	*Mugil cephlus* *Alestes baremose* *Hydrocynus* sp.	Low salt level after maturing phase; second has a high salt content and can be eaten after storing	Maturing phase: 15–20 days Storage phase: 2–3 months	Room temperature	[32,33]
Surstömming	Sweden	Whole fish	*Clupea harengus*	Brining	3–4 to 10–12 weeks	15–18 °C	[17,34,35]

### 2.1. Fish Sauces and Pastes

A variety of fermented fish products, from different regions of the world, with different fermentation parameters are sold (Table 1). Each of them has a distinct flavour, texture, and appearance, and may be consumed in various ways. Fish sauce is a popular condiment and seasoning in Asian countries and has gained popularity in the West. There are diverse types of fish sauce, from different geographical origins. The fish sauce can be either clear or turbid, depending on the degree of hydrolysis during fermentation, length of fermentation period, and separation process after fermentation [36]. Nampla from Thailand, budu from Malaysia, bakasang from Indonesia, and yu-lu from China are examples of fish sauces from Asian countries.

Nampla, also known as Thai fish sauce, is a clear brown liquid and one of the most exported fermented fish products from Thailand. *Stolephrous* spp., *Ristrelliger* spp., and *Cirrhinus* spp. are fishes commonly used in nampla’s production, where fermentation with salt or brine occurs up to 18 months, depending on the size of the fish [9]. The Thai Public Health Ministry classified fish sauce into three classes, which includes pure fish sauce, hydrolysed fish sauce, and diluted fish sauce. These classes are based on the production process, raw material, and quality of the fish sauce [9,20]. On the other hand, budu is a more turbid kind of fish sauce, with a brown-to-black colour. In general, the process of making budu is similar to that of nampla, which involves the fermentation of fish, such as *Stolephorus* spp., *Sardinella* spp., and *Decaterus macrosoma* with salt, and can take around 6–12 months [21]. After fermentation, the addition of flavourings, such as palm sugar, tamarind, and monosodium glutamate are added to the mixture, before boiling and filtering [22].

In North Sulawesi of Indonesia, bakasang is widely consumed; its preparation involves the fermentation of the viscera of *Katsuwonus pelamis* and small fish, or both [23,24]. Before fermentation, the fish gut is preconditioned in a warm place, where traditionally it is placed next to the source of heat in the house. This is to accommodate the natural fermentation, which occurs in the range of 30–50 °C [24]. It has unique flavours, with a light brown appearance, and more turbid and thicker consistency [23,24]. In China’s Southern and Eastern regions, yu-lu is a dark brown fish sauce regularly consumed as a condiment [25,26]. It involves the fermentation of small marine fish, mostly anchovies (*Engraulis japonicus*) and snakehead fish (*Channa asiatica*) [5]. Instead of warming the fish as in bakasang, yu-lu requires the pre-conditioning of the fish in salt in about a 1:3 salt-to-fish ratio. Like most fermentation, yu-lu is the product of the enzymes secreted by microorganisms, in this case, halotolerant and halophilic microorganisms due to the salt conditioning [25].

### 2.2. Whole Fermented Fish

In addition to fish sauce, fermented fish products come as a whole fish as in the original form. Among the many varieties of whole fermented fish around the world, examples include shidal (India), jeotgal (Korean), katsuobushi (Japan), feseekh (Egypt), and surstömming (Sweden). Although they are categorised under whole fermented fish, the appearance and flavour of the fermented fish still contrast with each other. Shidal is a fermented fish with strong odour and flavour, originating from the Northeast region of India. It is prepared from the fermentation of *Puntius* spp. and *Setipinna phasa*, without the addition of salt. The fish is then fermented in air-tight earthen containers in semi-open huts for about 3–5 months. The compressed appearance of shidal still retains the structure of the raw fish with some disintegration on the belly and caudal of the fish. Good quality shidal should progressively turn slightly brownish to dark brown, from its initial dull white state [27,28].

Next, Korean jeotgal consists of fermented seafoods, which can be categorised based on the types of main ingredients (seafood) and addition of seasoning [29]. Examples of jeotgal made from fish are myeolchijeot (anchovies), jeongeorijeot (sardine), chokijeot (croaker), euneojeot (*Coilia nasus*), and kongchi jeot (saury). Meanwhile, seasonings added to jeotgal can comprise only salt, or with the addition of Korean red peppers, soy sauce, and/or malted rice [29]. Jeotgal can be consumed by itself or as additives to enhance the flavours of other foods such as kimchi [30,37]. Next, in Japan, katsuobushi (dried bonito) is an essential food to the Japanese cuisine, where it is used for “dashi” stock [31]. Katsuobushi is usually produced using skipjack tuna (*Euthynnus pelamis* or *Katsuwonus pelamis*) through a repetition of moulding and sun-drying. Other types of fish also can be used as raw materials, such as eastern little tuna (*Euphonia affinis*), frigate mackerel (*Auxis rochei*), frigate tuna (*Auxis thazard*), and oriental bonito (*Sarda orientalis*) [5,6,31]. Thus, the form of pre-fermentation is called “arabushi”, whereas the ripened form after fermentation is “karebushi” [6]. The ripened and dried bonito has a cultivated layer of mould on its surface and a brownish appearance and hard exterior [31].

In Egypt, feseekh is a salted and fermented fish, which is consumed as an appetiser and main dish in feasts. Feseekh’s raw material can consist of either Bouri fish (*Mugil cephlus*) or pebbly fish (*Alestes baremose*). Tiger fish (*Hydrocynus* sp.) also can be used [5,33]. There are two varieties of feseekh available in Egypt; the first has a low salt level and may be eaten after maturing for 15–20 days, while the second has a high salt content and can be eaten after storing for 2–3 months [33]. The fermentation of feseekh involves a dry or wet salt process, and the final product with its unique sensory attribute is then sealed in containers for storage [32]. Finally, surstömming is a sour fermented Baltic herring (*Clupea harengus*) consumed in northern Sweden [34,35]. It has a distinctive strong sour and fizzy flavour, with a wine-coloured and swollen appearance [34]. Before fermentation, the process starts with the brining of fresh herring, before evisceration and another round of brining with weaker brine in barrels [35]. Notably, it is normal for cans of surstömming to swell or bulge, as the canning process does not require sterilisation and the fermentation continues in the can [17].

In brief, fermented fish is consumed as a condiment, seasoning, side dish, or even the main delicacy, all over the world. A diversity of fermented fish products can be found, with variations in the types of fish, additives such as salt and seasoning, as well as the fermentation process. These variations produce fermented fish products with distinguishable sensory attributes; these fish products are consumed as a source of nutrients, and the added benefits from the microbiota involved in fermentation. By understanding the traditional methods of fermented fish production, advancements can be made to enhance the efficiency and quality of the products. Therefore, in this study, we analyse the physicochemical and biochemical characteristics of the products, along with the microbiota involved in the fermentation process.

## 3. Health Benefits of Fermented Fish Products

**Figure 1:**
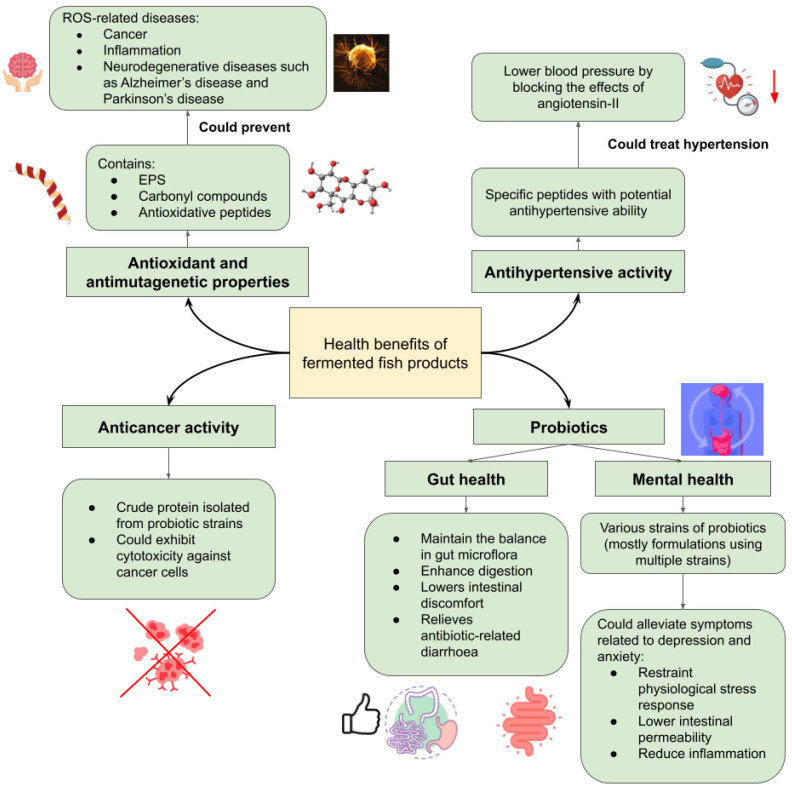
The health benefits of fermented fish products.

Fermented foods have various health benefits, which also depend on the microbiota, its by-product, and the raw material itself. As for fermented fish, its health benefits revolve around its antioxidant, antimutagenic, and anticancer properties; as well as probiotics for gut health and possible effects on mental health, and antihypertensive activity from the microbiota involved in the fermentation process (Figure 1).

### 3.1. Antioxidant and Antimutagenetic Properties

In aerobic organisms, the cellular metabolism generates products such as reactive oxygen species (ROS), free radicals, and reactive nitrogen species (RNS). These free radicals can cause oxidative stress to the body when there is an imbalance between the free radicals and antioxidant produced. This will lead to damage of biomolecules in the body, such as proteins, DNA, and membrane lipids [18,38]. Thus, it is vital to have an external source of antioxidants consumed in the daily diet, which fermented fish products can provide. Various studies have shown that fermented fish products can be a reliable source of antioxidants and have antimutagenic properties. Fermentation of fish causes the breakdown of protein, which releases specific sequences of peptides. These peptides are suggested to have anti-hypertensive and antioxidant properties [39]. To note, 2,2-diphenyl-1-picrylhydrazyl (DPPH), superoxide, hydroxyl, and 2,2′-azino-bis(3-ethylbenzothiazoline-6-sulphonic acid) diammonium salt (ABTS) are used as the measurement of the radical scavenging ability of the molecules in question [18,39,40,41]. During fermentation, whangseokeo-jeot (yellow corvina) had increased radical scavenging activity and reducing power that may have been caused by the Maillard reaction’s production of carbonyl compounds [42]. While another variety of jeotgal, myeolchi-jeot, showed 26.6 and 43.4% antimutagenic activity, when fermented for 6 and 12 months, respectively [29]. In a study of the antioxidant activity of ngari, a salt-fermented fish from Northeast India, potential antioxidant properties were found. However, their presence was dependent on the protein concentration and period of fermentation of ngari [39]. In another study on ngari, the probiotic isolate from ngari, *Enterococcus faecium* BDU7, was shown to excrete extra polysaccharide (EPS), which demonstrated significant radical scavenging ability [41]. Budu and pekasam also showed antioxidant properties from specific polypeptides obtained via fractionation of the fermented fish extracts [18,40]. The budu extract was deproteinised to acquire the antioxidative peptides from *Ilisha Melastoma* (anchovy). The peptides from the budu extract, LDDPVFIH and VAAGRTDAGVH, showed radical scavenging activities for DPPH and ABTS radicals [18]. Similarly, antioxidative properties were displayed by IAEVFLITDPK and AIPPHPYP peptides from fractionation of pekasam [40]. The consumption of the specific polypeptides from the fermented fish products was shown to prevent ROS-related chronic diseases, such as cancer, inflammation, and neurodegenerative diseases such as Alzheimer’s disease and Parkinson’s disease [43], owing to antioxidant activities (Figure 1).

### 3.2. Anticancer Activity

Utonga-kupsu, which is a type of fermented fish produced in the Northeast region of India by the indigenous Manipuri people, has shown promising anticancer activity [44]. The cytotoxicity against cancer cell lines, which involves HeLa and HT-29, has been observed of the crude protein isolated from UK3 (*Staphylococcus* spp.), UK10, and UK12 (*Staphylococcus piscifermentans*). When the cell reaches the primary leukocyte stage, probiotic bacteria isolated from utonga-kupsu begin to impede the growth of mammalian cells. This contributes to the anticancer activity of the probiotic isolates (Figure 1). However, the bacteria did not exhibit cytotoxicity to the normal lung cell L-132, which is vital for it to be deemed safe in a biopharmaceutical application [44].

### 3.3. Probiotics for Gut Health and Possible Effects on Mental Health

The fermentation of fish requires a consortium of microorganisms, and some of them are of the probiotics strain. Probiotics, which have become more popular, can be sourced from fermented food such as yoghurt, kefir, kimchi, and fermented fish. The characterisation of probiotics isolated from fermented fish can be determined by investigating its gastric acid, bile salt and phenol tolerance, thermotolerance, cell adhesion using intestinal epithelial cells or biofilm production, and antibacterial and antibiotic sensitivity, as well as lysosomal activity [44,45]. The most common probiotic isolated from fermented fish is lactic acid bacteria (LAB) [29]. Resistance to artificial gastric and bile juice and antimicrobial activity to *Listeria monocytogenes* was observed in commercial Korean jeotgals such as ojingeojeot (squid), koltugijeot (small squid), and myeolchijeot (anchovy) [29,46]. *Lactobacillus brevis* strain LAP2 has also been successfully isolated from hentak, which is a fermented fish from Northeast India, and subsequently characterised [47]. Other than LAB, other bacteria such as *Staphylococcus piscifermentans*, *S*. *condimenti*, and *S*. *carnosus* were identified as probiotics, and isolated from utonga-kupsu [44]. Probiotics are an important part of the diet, as they benefit gut health (Figure 1) by maintaining the balance in the gut’s microflora, enhance digestion, lower intestinal discomfort, and aid in relieving antibiotic-related diarrhoea [47,48]. Thus, fermented fish products can be a reliable source of probiotics.

Worthy of attention is the claim that probiotics can alleviate the effects of anxiety and depression. It is common for mental illnesses such as depression and anxiety to coexist with digestive issues, indicating a bidirectional relationship between gut health and mental health [49,50]. Mechanisms such as neuroinflammation, inflammation, intestinal permeability, and disturbances in the hypothalamic–pituitary–adrenal (HPA) axis have been suggested to explain this comorbidity [50,51]. Probiotics from fermented food such as fermented fish have been shown to have positive effects on the mechanisms (Figure 1), whereby they help to restrain the physiological stress response and lower intestinal permeability, which consequently inhibits endotoxemia and alleviates inflammation [51]. Therefore, probiotics may be a potential treatment that focuses on the gut microbiota for the prevention and treatment of prevalent mental health illnesses [52]. Nonetheless, the effect so far is mostly strain-specific, as the studies conducted applied various strains with mostly formulations using multiple strains and dosages. Thus, more research needs to be done, so that a judgement can be inferred on the application of probiotics in the mental health sector.

### 3.4. Antihypertensive Activity

Ngari’s antihypertensive ability has been studied by identifying its angiotensin-I converting enzyme (ACE) inhibitory activity. Similar to its antioxidant activity, proteolysis during fermentation by microorganisms cause the release of the specific peptides (Figure 1). These peptides are potentially antihypertensive, which is affected by the concentration of the peptides and fermentation period [39]. Thus, fermented fish can be a promising source of antihypertensive peptides for hypertension treatment. ACE inhibitory effects were found to lower blood pressure by blocking the effects of angiotensin-II [53].

The health benefits mentioned were mostly supplementary to the prevention or alleviation of disease symptoms. Although fermented fish products have not been proven to effectively cure diseases, they are widely helpful in maintaining the physical and possibly mental health of individuals. As such, studies should ensue in pursuit of proper medical applications of fermented fish products. For instance, fish fermentation methodologies could be used to extract beneficial contents, such as EPS, carbonyl compounds, and antioxidative peptides in prevention of ROS-related diseases (Figure 1). With this in mind, medically approved substances produced from fish fermentation could greatly aid in the availability of supplements and medication. As such, the economical aspect of this direction should also be assessed and improved with other factors in fish fermentation, especially the physicochemical and biochemical characteristics, which will be discussed later in this review.

## 4. Ingredients Used in Fermented Fish and Fish Products

**Figure 2:**
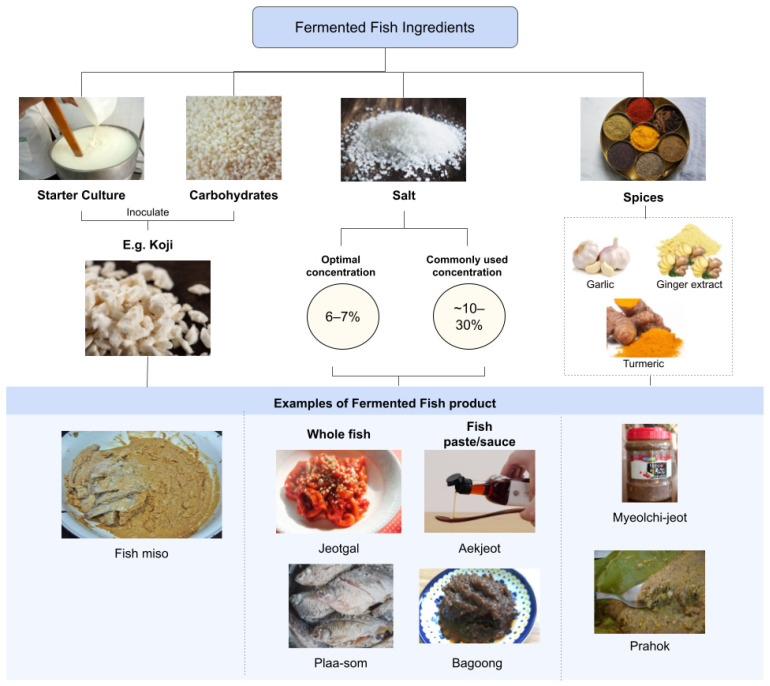
Fermented fish ingredients and their respective product example.

As a delicate process, fermentation requires certain conditions to be met to ensure the quality of fermented fish. Naturally, additives and ingredients are included in the recipes of fermented fish to maintain the ideal parameters for fermentation to occur. Starter cultures, carbohydrates, salt, and spices are added to fish depending on the culture to aid in the fermentation and quality of the product (Figure 2). These ingredients are designed to cater towards the optimal conditions of the microorganisms and enzymes that are responsible for the main reactions of fermentation.

### 4.1. Starter Culture

Since microorganisms and enzymes are bound to be involved in the fermentation of fish, adding them to the process as ingredients can speed up the process. In the case of enzyme fermentation, commercial enzymes such as papain, bromelain, trypsin, pepsin, and chymotrypsin are added to fish to enhance the fermentation process and ensure the quality of the fermented fish products [10]. Similarly, products that involve microbial fermentation often include starter cultures as part of the ingredients to accelerate the fermentation process. In a previous study, five different fish fermentation products using mixed cultures were compared with a spontaneously fermented fish, in which all mixed cultures proved to hasten the growth of lactic acid bacteria and eliminate Enterobacteriaceae from the product compared with spontaneous fermentation [54]. It was found that starter cultures of *Staphylococcus xylosus* with *S.carnosus*, and *S. xylosus*, *S. carnosus*, *Pediococcus pentosaceus* with *P. lactis* could be effective for fermented fish-chili paste [54].

Moreover, the use of more than one type of microorganism as starter cultures seem to be the better option in fish fermentation. This is because while one microorganism could potentially enhance the fermentation process in fish, it might not be able to effectively prevent the growth of pathogenic microorganisms at the same time. For plaa-som, a freshwater fermented fish from Thailand, single strains of either *Lactobacillus plantarum* IFRPD P15 or *Lactobacillus reuteri* IFRPD P17 were unable to both decrease the pH for better fermentation and control the growth of harmful bacteria [55]. The ideal effects of the starter culture could only be seen when both microbes are included as *L. plantarum* IFRPD P15 prevents the growth of harmful bacteria while *L. reuteri* IFRPD P17 maintains optimal pH for better fermentation and safety for consumption [55]. This is also seen in a fermented fish in China called suan yu, where not only did mixed cultures successfully lower the pH and inhibit unwanted bacterial growth, but also prevented the accumulation of total volatile base nitrogen (TVBN) and thiobarbituric acid reactive substances (TBARS) in fermented fish [56]. According to the study, *L. plantarum*, *S. xylosus*, *S. cerevisiae*, and *P. pentosaceus* were a few of the microbes involved in the starter cultures [56]. Thus, it can be noted that fish fermentation is more efficient and effective with the starter culture added.

### 4.2. Salt

Salt is one of the major ingredients added to fermented fish. Salt provides additional flavour to the fermented fish and reduces the water activity of fish, thus preventing unwanted growth of bacteria [29]. Salt is a commonly used ingredient for traditionally fermented fish, which can be exemplified with the Korean jeotgal and aekjeot, whereby only salt is added to fish to be fermented [29]. Fish salting is especially beneficial if the fermenting microbe is salt tolerant or halophilic, as seen in the fermentation of myeongtae sikhae (Alaska pollock), whereby *Lactobacillus sakei* flourished after fermentation in a salted condition while other bacteria were prevented from growing [29]. The optimal salt concentration for lactic acid bacteria to ferment fish is about 6–7%; higher salt concentrations would lead to the growth of certain strains of *Staphylococcus* spp., which may be harmful when consumed, like *S. aureus* [57]. This is seen in plaa-som, in which fermentation was faster in lower salt concentrations as the pH decreased, aiding in lactic acid bacteria growth [57]. However, higher salt concentrations within ~10–30% are commonly used. Although these recipes have been traditionally passed down, noteworthy is that overly salted fermented fish products are potentially harmful due to the higher risk of contamination by halophiles, especially since most halophiles are able to proliferate in low water activity (0.755) [58]. Though highly salted fermented fish have lower water activities compared with the slightly salted fish, the higher salt concentrations are still unable to prevent the proliferation of certain strains of harmful microbes. Similarly, salt is also used in fermented fish paste (Figure 2), which requires a fermentation period longer than fermented fish but shorter than fish sauce production (8–32 days). As an example, bagoong is a fish paste produced in the Philippines by mixing *Stolephorus* spp., *Sardinella* spp., and *Decapterus* spp. with either whole or ground fish roe and small shrimp (*Atya* spp. or its roe), with 25% salt [59]. In comparison, plaa-som utilised less salt than bagoong, although both are considered highly salted (~10–30% salt). From another perspective, this comparison shows the relationship among the salt concentration needed, fermentation period, and texture of fermented fish products. Overall, noteworthy is that while the addition of salt can be beneficial to the growth of fermenting microbes, the amount of salt added should be controlled to prevent oversalting to prevent growth of harmful bacteria.

### 4.3. Carbohydrates

Carbohydrates are required for lactic acid fermentation in fish at low salt concentrations (less than 20%) [60]. Rice, millet, flour, sugar, and sometimes garlic are added to the fish as a carbohydrate substrate for the lactic acid bacteria to ferment, as fish contains too little carbohydrate content to act as a substrate for fermentation [57,60]. Previous studies have suggested that carbohydrates mainly prevent the high buffering capacity of fish to achieve lower pH values favouring lactic acid bacteria growth [57]. The lowering of the pH by the production of lactic acids in fish also induces gelation. Cross-links form in the myofibrillar proteins in fish, causing the texture in fermented fish to change [61]. In that sense, the addition of carbohydrates aid in the texture qualities of fermented fish as well. Similarly, carbohydrates are used in fermented fish paste production (Figure 2). This can be seen in Japanese cultures where koji, *Aspergillus oryzae*-inoculated rice malt, is used as a starter culture for fermented fish miso production [11].

### 4.4. Spices and Seasoning

To enhance the flavours and aroma of fermented fish, spices are included in the recipe according to cultural preferences. For instance, Cambodian fermented fish are well seasoned with cloves, cinnamon, star anise, nutmeg, cardamom, ginger, turmeric, and many more spices to create flavours and colours that peak the consumer’s interest [62]. In addition to the flavour aspects, spices can also contribute to the fermentation process. It was noticed that garlic (along with glycine) in fish fermentation helps to decrease amino acid decarboxylation, especially in reducing cadaverine and tyramine contents in myeolchijeot (fermented anchovies) [60]. Moreover, garlic has antimicrobial properties that prevent the growth of unwanted bacteria (e.g., *Salmonella typhimurium*, *Escherichia coli*, *Bacillus cereus*, *Staphylococcus aureus*, and *Listeria monocytogenes*), in addition to its enhancement of lactic acid bacteria growth, making garlic a very common ingredient in fish fermentation [57,63]. As another example, ginger extract has been found to be a potent antioxidant, which is needed in non-fatty fishes like tilapia in fermentation [64]. This is to preserve the valuable lipid contents from the raw fish from being oxidated during the fermentation process.

In short, the ingredients added to fish for fermentation usually help to enhance the flavours of the fermented fish products and aid in the fermentation process, from acting as a source of nutrients for the fermenting microbes to idealising the environment for microbial activity. While most studies mentioned the ingredients of fermented fish products, the benefits of the specific ingredients were not as emphasised in most, especially spices. As such, further studies could be conducted to observe whether additional ingredients in fermented fish play any part in fermentation other than to provide flavour.

## 5. Biochemical and Physiochemical Characteristics of Fermented Fish

Fermentation changes the biochemical composition of fish meat drastically as microorganisms and enzymes degrade components in fish. The quality of fermented fish can be determined from analysing the proximate composition of fermented fish as well as certain physicochemical changes such as lipid oxidation, proteolysis, pH, and water activity (a_w_) in fish fermentation, especially to determine the sensory attributes of the product. Not only does understanding the biochemical and physicochemical characteristics of fermented fish ease its production, the preferability of the products along with their safety in consumption are also determined.

### 5.1. Biochemical Characteristics of Fermented Fish

#### 5.1.1. Proximate Composition of Fermented Fish

The proximate composition of fermented fish consists of moisture, lipids, proteins, ash, carbohydrates, and minerals. The different amounts of these substances in fermented fish constitutes the general biochemical characteristic of the products. Thus, these contents are studied attentively for the sake of understanding the safety and health aspects of fermented fish and improving the production of fermented fish.

#### 5.1.2. Moisture

Most studies have found that fermentation causes the moisture content in fermented fish to increase over time [65,66]. This increase in moisture content was stated to be due to absorption of water from the environment and the release of volatile contents from fermentation reactions in fish [66]. On the other hand, there have also been studies that mention the decrease in moisture content in fish after fermentation, caused by the osmotic migration of salt and water, with moisture drawn out and salt entering the fish during fermentation [67]. This concept was also supported by another study, concluding that more moisture flowed out of the fish than in during fermentation [68]. This contrast can be explained with the different types of water in fermented fish that contribute to the moisture aspect in its proximate composition. In a study about the moisture content of fermented stinky mandarin fish (*Siniperca chuatsi*), varying water-related aspects were taken into consideration. Notably, the water content decreased from 77.72 to 55.50%, while the water-holding capacity increased from 85.63 to 98.81% [69]. This is due to the existence of bound water, intermediate water, and free water. From the study, it was found that fermented stinky mandarin fish lost a significant amount of free water but retained most bound and intermediate water [69]. While theoretically, the free water would be lost and bound to the molecules and the intermediate water would be retained, the actual ratio of water loss affected by the osmotic activities involved would require further studies using advanced technologies. As important as it is to specify the type of water content in the context, the targeted definition of the term “moisture” should also be considered. As previously mentioned, the increase in moisture during fermentation could indicate the degradation process in fermentation [66] while the decrease in moisture content could be referring to the loss of free water due to osmosis [67,68], both of which are relevant in the topic of moisture in fermented fish. Of course, the moisture content in fermented fish also highly depends on the type of fish used as well as the method of fermentation (Table 2).

#### 5.1.3. Lipids

Lipid contents in fish are seen to be generally healthier than in meats as most fats in fish are unsaturated. The lipid content is overall generally lower compared with protein content (Table 2), making it a more preferable protein choice for those seeking low fat diets. Ngari and hentak are the two most preferred fermented fish in North-Eastern India. From the results of the fatty acids profile in ngari, palmitic acid (C16:0) was found to be dominant; whereas stearic acid (C18:0) was dominant in the case of hentak. This finding is corroborated by the study of [75], which analysed the fatty acids composition in traditional salted fermented fish in Sudan. However, the ratio of saturated fatty acids (SFA), monounsaturated fatty acids (MUFA), and polyunsaturated fatty acids (PUFA) changes with the fermentation process. There are several contradicting results on the levels of SFA, MUFA, and PUFA in fermented fish [76], and only docosahexaenoic acid (DHA) has been reported to increase in most studies on fish fermentation. While one study reported a decrease in fatty acids (FA) and increase in SFAs [77], another reported increased SFAs and MUFAs but decreased PUFAs [78]. Most recent studies asserted declined levels of SFAs and MUFAs and a rapid increase in PUFAs. The discrepancy in the results might be contributed by the type of fermented fish, fermentation, conditions, the addition of different additives [76], and lipid/fat content in the fish body. It should be noted that fatty fish have a higher percentage of fat compared with lean fish. The fatty acid composition of the muscle cell membranes is a crucial factor in determining membrane stability because initiation of lipid oxidation occurs in the phospholipid bilayer of the muscle membrane [79]. A thorough investigation is warranted to determine the mechanism that governs different fatty acid levels and lipid oxidation during fish fermentation in the future. As for the changes of lipid contents in fermented fish products, there is no distinct trend based on the examples used in Table 2. For example, adjuevan samples showed a slight decrease in lipid after fermentation, yet shidol presented an increase in lipid content post-fermentation. Therefore, more research can be conducted to investigate the parameters that affect lipid content in fermented fish products.

#### 5.1.4. Protein

With fish being a rich source of protein, it is crucial to understand the effects of fermentation on one of the most valuable properties of fish consumption (Table 2). It was previously found that surströmming, a traditional Swedish fermented herring, contains 11.8% protein [5]. The protein content differs depending on the type of fish used to make the fermented product. Otherwise, fermentation does not change the total protein content in the fermented fish product. This is seen in another study whereby the 23% of protein content in raw mackerel showed no notable fluctuations throughout the fermentation process [68]. However, an increase in protein content in the rice mixture (2.3–8.8%) used in the fermentation process concluded that a small amount of protein from the mackerel flowed out from the fish itself into the rice mixture surrounding the fish during fermentation [68]. While this does not change the overall content of protein in this fermented fish product, it is an important factor to consider when fermenting fish with carbohydrates added to the fish.

As a food, the involvement of amino acids responsible for the “deliciousness” of fermented fish products are also studied, albeit are less common than research on the health aspects of the protein contents in fermented fish. Delicious amino acids (DAAs), otherwise known as umami amino acids, is a category that represents amino acids that contribute to the pleasant tastes and flavours of foods. Glutamate, aspartate, glycine, and alanine are commonly considered delicious amino acids (DAAs) in previous studies [80,81]. It was found that glutamate and aspartate, both responsible for the umami taste in foods, were found in fermented fish products [82]. For fermented anchovy sauce, all four DAAs were found to be present and are major contributors to the product’s taste [80]. However, it was also found that glutamate was the main contributing amino acid to umami tastes in fermented fish sauce [82]. The contents of DAAs in fermented fish changed with fermentation time as well [82]. That being said, fermented fish products are high in DAAs necessary for the enjoyment of consumers, although adjustments in specific amino acids and their amounts could be made. In addition to these sensory observations, different sensory attributes need to be considered in the fermentation process, which will be discussed further in this review, as not only the taste attracts consumers’ attention.

#### 5.1.5. Ash

Ash content, otherwise known as inorganic substances, found in fermented fish vary according to the nature of the fish itself. This is observed in the differences in ash content between hentak and ngari (Table 2), fermented fish products from Manipur. Hentak has higher ash content compared to ngari due to the scaly and bony nature of Indian flying barbs, the fish used to make hentaak [72]. Additionally, the high ash content could also be explained by the presence of alocasia from the preparation of hentak [72]. Furthermore, different types of fermentation methods cause the ash content to fluctuate as well. Looking a Table 2, momone and jambal roti had higher ash contents compared with the other fermented fish products because both products are salted. Added salt seeps into the fish meat during fermentation and this ultimately increases the ash content in the final product [71].

#### 5.1.6. Carbohydrates

In proximate composition analysis of fish, carbohydrates are usually assumed to be negligible because they are typically present in a very small percentage (<0.5%) [83]. Nevertheless, carbohydrates should be taken note of in fermented fish, specifically the ones that include additional carbohydrates. As mentioned previously, rice, flour, sugar, and other types of carbohydrates are sometimes added to fish as a source of energy for microbial fermentation. One study observed that fermented fish paste generally comprises 1.10–24.19% carbohydrates [84]. The large range could indicate different fermentation methods for the fish pastes used in the study. On the other hand, the carbohydrate contents of tungtap, a fermented fish from the Meghalaya tribe in Northeast India, decreased from 0.45% to 0.3% after being fermented [85]. This decrease meant that the carbohydrates were being used up by the microbes present in the fish during fermentation [85]. Studies can be done to investigate whether the addition of carbohydrates is able to accelerate the fermentation process. In conclusion, while carbohydrate content is not the main focus in the discussions of fermented fish compositions, it needs to be further studied to fully understand the microbial activities during fermentation.

#### 5.1.7. Minerals

While minerals exist in smaller amounts in foods, their presence is significant for the health of consumers. Fermented fish has a higher content of calcium, potassium, sodium, and magnesium while iron, zinc, and copper were reduced [86]. Studies have found that not removing pin bones from the fish flesh prior to fermentation could be the cause of the higher calcium contents in fermented fish products such as ngari, hentak and telesech [86,87]. Similarly, calcium and phosphorus charted higher levels in fermented fish compared with fresh fish in a study focusing on fermented fishes in the Lower Mekong Basin [88]. The fermentation and degradation of bones played a huge part in the calcium content, which heavily relies on the cultural preferences of fish butchering prior to the fermentation processes [88]. The study also mentioned that less calcium was found in fermented fish that had the bones and heads of fish removed prior to the fermentation. In that sense, calcium and phosphorus were found to be the most prominent minerals in fermented fish (Table 2), with the consideration that the bones may play a big part in their contributions.

### 5.2. Physicochemical Characteristics of Fermented Fish

During fish fermentation, three main reactions take place: proteolysis, lipid oxidation, and lipolysis. Protein and lipid components in fish are degraded into smaller polypeptides and amino acids, and fatty acids, respectively, by endogenous enzymes in the muscles together with microbial enzymes. Additionally, the pH changes and a_w_ in the fish flesh tremendously affect the quality of fermented fish, especially in terms of microbial growth. These physicochemical changes during fermentation decide if the final product is able to achieve the desired sensory attributes.

#### 5.2.1. Lipolysis and Lipid Oxidation

There are distinct differences between lipolysis and lipid oxidation in fish fermentation mechanisms. In short, lipolysis helps to release free fatty acids (FFAs) from fish while lipid oxidation converts the FFAs into hydroperoxides, which can then be further decomposed into flavour compounds (e.g., methyl ketones) or react with amino acids to form odour compounds (e.g., esters) [89]. The substrates from these reactions are mostly involved in the aromatic aspects of fermented fish. Thus, they contribute to the flavours and odours of the product, making lipid degradation a crucial step in fish fermentation. Thus, lipolysis and lipid oxidation go hand in hand and are quite inseparable from each other if quality flavour and odour is to be acquired from fish fermentation. However, some studies have claimed that lipolysis and lipid oxidation have no relation with each other due to the differences in the parameters and substances involved [90,91]. These claims resulted from the distinction between parameters in lipolysis and lipid oxidation instead of the overall theoretical lipid degradation process. Regardless, lipolysis and lipid oxidation must occur during fish fermentation.

**Figure 3:**
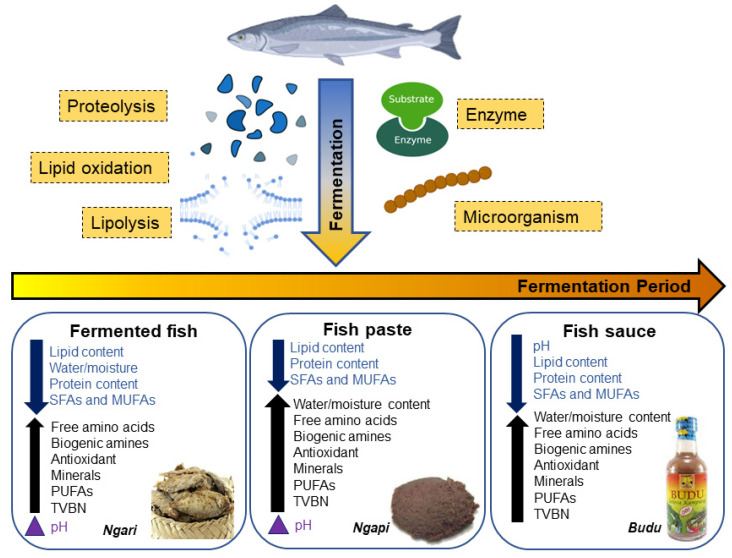
Biochemical reactions and changes during fish fermentation.

Lipolysis is generally catalysed by lipases found in the adipose tissues (neutral lipase, acid lipase and phospholipase) and muscles of the fermented fish [89,92]. Different types of lipolytic enzymes cause the release of different FFAs, with neutral lipases having the highest activity and acid lipase having the lowest activity [92]. This result contradicted the findings from another study stating that all three types of lipases decreased in their activity during fermentation as the concentration of lipases were lower in the final fermented fish product than when the fish was raw, with phospholipase having the lowest activity while acid lipase had higher activities [90]. Thus, there is no general trend in the lipase activities, as it inconsistently increased and decreased throughout the fish fermentation process. Notably, a previous study has found that bacterial lipases also help in degrading the lipids in fish into FFAs [93]. Nine different bacterial strains that produced extracellular lipases (categorised into *Corynebacterium*, *Virgibacillus*, *Oceanobacillus* and *Bacillus* genera) were isolated from Thai fermented fish products (koey-pla, kee-dee and tai-pla), indicating that bacterial enzymes contribute to lipolysis of fish during fermentation. This was concluded from the greater reduction of myristic acid, palmitic acid, and stearic acid in inoculated samples compared with raw samples in the study [89]. In brief, lipase is responsible in converting lipids in fish into FFAs via fermentation (Figure 3). Thus, optimisation of the lipase production by microorganisms can be studied to increase FFAs in fermented fish products.

Lipid oxidation can be categorised into autooxidation and enzymatic oxidation, in which autooxidation occurs naturally and enzymatic oxidation mostly occurs with the help of lipoxygenase (LOX) [92]. Free radicals (e.g., peroxyl radicals, alkoxy radical) are often the product of these reactions, which forms other hydrocarbon compounds through different metabolic pathways such as alcohol and aldehyde along with the formation of acids [92]. Lipid oxidation can also be categorised further into primary oxidation and secondary oxidation, both producing different compounds [92]. Endogenous and exogenous enzymes from the fish and microbes also contribute to lipid oxidation aside from LOX as the major contributor. In a previous study, it was found that LOX activity was initially higher in fish fermentation. Then, it decreased in both high salt and low salt conditions due to the inactivation of hydroperoxides in the latter stages of fish fermentation [90]. It was also proposed that the presence of salt could activate LOX, making its activity higher during the initial stages of fermentation [90]. Thiobarbituric acid reactive substances (TBARS) and peroxide values (POV) are significant indicators of secondary lipid oxidation products. TBARS and POV increase in the initial stages of fish fermentation and decrease at the latter stages, similar to the LOX activity [90]. However, this contradicts the results from another study on som-fug, a salted fermented fish product from Thailand, stating that TBARS value was the highest in the fermented fish stored for a longer period of time [94]. There might be a difference in lipid oxidation during the fermentation and storage stages, whereby lipid oxidation continues to occur during storage of the fermented fish products. Nevertheless, further studies should be conducted to understand the lipid degradation reactions in fish fermentation. Not only is the relationship between lipolysis and lipid oxidation still unclear and ununiform among researchers, the diversity of fermented fish products and their fermentation methods greatly differ the lipid degradation processes from one to another.

#### 5.2.2. Proteolysis

Proteolysis is one of the few key processes in fermentation that enhances the products’ flavours and sensory attributes. Similar to lipid degradation, proteolysis occurs via endogenous and exogenous enzymes from the fish and microbes, respectively [95]. In the same sense as lipids with FFAs, the amino acids released from the proteolytic processes are important flavour components comprised in the value of fermented fish. The common amino acids that were found to increase in fermented fish are non-essential amino acids such as glutamic acid, aspartic acid, and glycine [86,96]. Non-essential amino acids were found to contribute to the taste attributes of fermented fish. By lowering the pH of fermented fish, proteolysis is induced and this changes the composition in the fish [95]. Protein denaturation caused by low pH eases proteolysis, which in turn more easily releases the desired amino acids, thus decreasing the protein content in fermented fish (Figure 3). Endogenous cathepsins B, H, L, and D from fish mainly catalyse the proteolytic process [95]. In fish sauce production, the key proteases are trypsin and cathepsin B assisted by enzymes from the microorganisms. Other enzymes have been implicated in the breakdown of polypeptides to amino acids such as aminopeptidase, which showed high activity during sardine fermentation [97]. Glutamic acid and aspartic acid were the most prominently found amino acids from a study about suan yu [95]. At the same time, histidine was also one of the amino acids found in higher concentrations during fermentation. While amino acids are useful in fermented fish for enhancing sensory attributes, harmful biogenic amines like histamine can be derived from the amino acids [98]. Therefore, the proteolytic processes in fermented fish should be studied in more detail for better control of the substrates.

#### 5.2.3. pH

The main step in fish fermentation is a combination of low pH and organic acid [99]. Generally, during fermentation, pH values will decrease slightly and fluctuate between 6.47 and 6.58 [100]. The decrease in pH is most probably due to lactic acid accumulation by LAB. The pH values in 18 different fermented fish products used in Asia were reported to range from 4.3 to 7.8 [96], indicating that most of the fermented fish in Asia are alkaline. In alkaline fish, the hydrolysis of proteins into peptides and amino acids causes the release of ammonia, which then raises the final pH [96]. High pH and ammonia control the growth of several dominant bacteria, and this allows the anaerobic breakdown of proteins to occur and finally release amine compounds. Suan yu, a traditional fermented fish in China had a pH value of 6.0 at the first 10 day’s fermentation and when a starter culture containing *Lactobacillus plantarum* was used; the pH significantly decreased to 4.83 [56], further implying the necessity of starter cultures in fish fermentation. In some studies, the pH is slightly higher due to the production of total volatile nitrogenous compounds, and their accumulation in the fish because of the fermentation process. In the case of fermented fish pastes, some have lower pH while in some others, the pH values are increased [101]. This increase in pH is also seen in fermented whole fishes and, as seen in Figure 3, biogenic amines increase when pH increases in the fermented products, suggesting that biogenic amines or lower salt concentrations during fermentation caused the pH to increase [102]. While that may be, lower pH is usually preferred in fermented fish as high acidity creates an antibacterial effect, and this indicates that the product is safe to eat.

#### 5.2.4. Water Activity

Water activity (a_w_) helps to determine whether microorganisms will be able to thrive in an environment of a certain food, depending on how much water is present for the microorganism to sustain itself and multiply. Usually, lower a_w_ accounts for longer shelf lives because most microorganisms are unable to proliferate under low water activity conditions. However, a certain amount of a_w_ is still required in fermented fish because of the roles of certain microbes and enzymes in fermentation. Studies found that a_w_ values of 0.65–0.87, or generally 0.7 and above, are able to sustain the necessary microbes in fermentation and inhibit growth of unwanted bacteria in fermented fish product [103,104]. In terms of the types of products, the a_w_ of fermented whole fish decreased while the values in fermented fish paste and sauce increased (Figure 3). While it may be wise to manipulate the external factors of fermentation such as the salt content, pH, and concentration of a starter culture to achieve the preferred range of a_w_, neither of the mentioned factors had any correlation to the a_w_ in fermented fish [103]. Needless to say, when opting for better quality microbial characteristics in fermented fish, a_w_ is the lesser concerned aspect in view of the more dominant factors like pH, salinity, and so on.

#### 5.2.5. Colour

With the physicochemical changes occurring in fish as it ferments, the colour aspect of the product is a major concerning factor for sellers as it affects the overall consumer acceptability. The colour of fermented fish is analysed by using the L*, a* and b* of the CIE colour model. L* indicates the lightness of the product, a* the redness/greenness, and b* the yellowness/blueness with higher values of a* and b* implying more redness and yellowness, respectively. The colour of som-fug was found to fade from a greyish-white or reddish-brown colour to a pinkish colour after a couple of days into the fermentation, caused by the denaturation of proteins and pigments in low pH conditions [61]. The ingredients and fermentation methods also have a big influence on the outcome of the colour of the fermented fish, although it was noted to generally turn lighter in colour, or for the L* values to increase with time. From another perspective, the fermentation of fish is reported to have minimal to no effect on colour [105]. However, the study that concluded this focused on the consumer attitudes instead of the physicochemical characteristics of fermented fish. The results charted 6.3 for fermented *Heterotis niloticus* and 5.8 for the unfermented fish on a 9-point Hedonic scale [105]. Hedonic scales are often used to measure the preferences of the panellists involved in studies regarding sensory characteristics of fermented fish products. The scales range from 5 to 9 points depending on the scope of the study, with the lowest number on the scale as the poorest rating and the highest number as the best [61,106,107]. Although the physicochemical characteristics of colour were not analysed, the colour of raw and fermented fish was visually indifferent from each other, which can be considered in future studies on the colour of fermented fish. In another study, spleen was added to fermented fish sauces and the colour of the final product was assessed. The addition of spleen seemed to increase the a* and b* values while decreasing the L* values at the same time [108]. It was also noted that greater salt concentrations caused the colour intensity to increase as well during fermentation. This was explained to be caused by the low molecular weight compounds and presence of high molecular weight melanoidins formed in the fish during fermentation as proteins and lipids are being degraded [108]. Furthermore, Maillard reactions took place in the fermented fish, explaining the increase in a* values as the fish browns. The Maillard reaction usually occurs from the impact of higher temperatures, as demonstrated in the drying of a Thai fermented fish dip, mentioned as a non-enzymatic browning process that increases the a* value [109]. Nevertheless, the colour changes in fermented fish products are, albeit slight, still an essential factor to consider when choosing a fermentation method for the fish.

## 6. Sensory Characteristics of Fermented Fish

**Figure 4:**
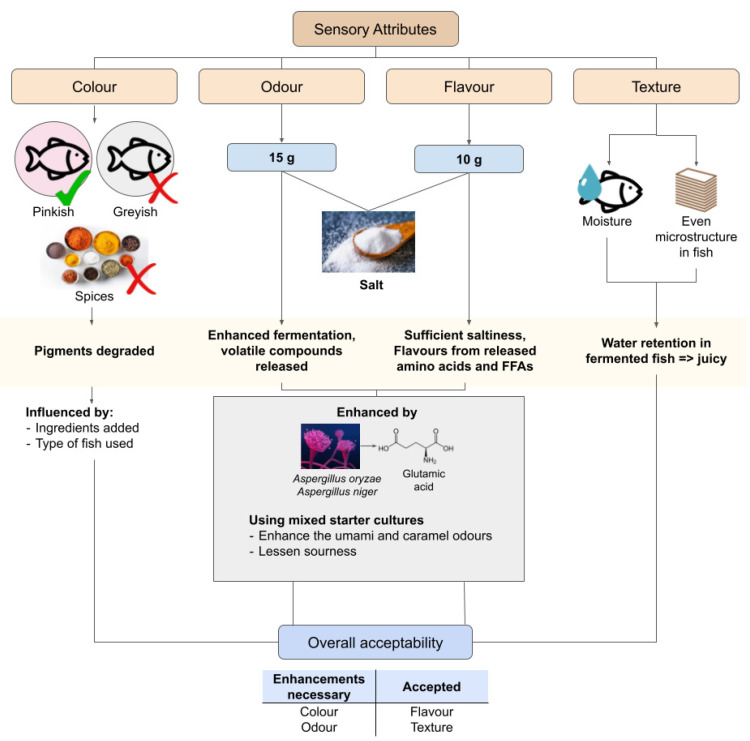
Summary of the sensory attributes of fermented fish in relation to their overall acceptability.

Sensory attributes are necessarily studied in fermented fish to understand the preferences of people towards fermented fish products. The parameters of the human senses assessed for this attribute are mostly qualitative such as colour, texture, odour, flavour, and overall acceptability (Figure 4). Usually, a number of willing participants, otherwise known as panellists, are selected to assess the fermented fish products according to these parameters and grade them according to a scale set by the researcher [61,106,107].

Thus, the results of each study might differ depending on the region in which the research was conducted, especially when cultural preferences highly diversify the sensory preferences of people. The preferences in cross-cultural settings towards a particular food differs slightly, as seen in a previous study in which people from the United States had slightly different opinions towards the flavour in a Korean dish compared with those from Korea [110]. This situation applies to fermented fish products as well, with the practices for preparing the fermented fish products differing between cultures and regions. Colour-wise, slightly pinkish fermented fish seem to be preferred over the ones that looked creamy and had black spots of unevenly grounded ingredients; and greyish fermented fish was the least preferred [61]. While all of the samples were the same type of product (som-fug) from different brands, the colour differences were likely caused by the varying types of fish used and ingredients added to the fermented fish products [61]. The added ingredients also influence the colour preferences of fermented fish to a certain degree. Notably, fermented fish pre-soaked in spices were less preferred than non-spiced fermented fish in terms of their appearance and colour [106]. Another notable result from this study is that the colour of fresh fish was the most liked compared to the ones fermented with 15 g salt and 10 g salt, likely due to the degradation of pigments mentioned previously [61,106]. Nevertheless, this proves that the colour attributes can be changed by adding ingredients to the fermented fish aside from using different types of fish (Figure 4).

Similar results were recorded for odour aspects of the fermented fish products in the study, in which fresh fish was more preferred compared with fish fermented in 15 g and 10 g salt (Figure 4), regardless of the presence of spices [106]. However, this pattern changed in the saltiness and flavour aspects, where the fermented fish fermented with 10 g of salt were more preferred than the ones fermented with 15 g salt as well as fresh fish [106]. This would mean that although fermented fish is less attractive in colour and odour, the saltiness and flavour of the fermented fish are what attracts people to consume it. However, studies have claimed that using mixed starter cultures can improve the odour of fermented fish products. It was found that a mix of *Aspergillus oryzae* koji and *Aspergillus niger* koji was able to enhance the umami and caramel odours in fermented fish while lessening the sourness and ammonia in the products [107]. Glutamic acid gives off an umami flavour [95]; and the mixed koji was able to increase its concentration in fermented fish up to 28.43% [107]. Thus, colour and odour, which are usually less attractive to consumers, can be enhanced.

As for the texture, it was reported that people felt that the textures in fish did not change significantly before and after fermentation [105]. However, the texture preference between different fermented fish products still exists. Products that had less moisture content or more water that osmotically flowed out of the fish were generally less liked, as was the uneven network of the microstructure in the fish [61]. This accounts for the juiciness of the fermented fish and how pleasurable it is to chew on the products (Figure 4). As such, the texture of fermented fish can be enhanced by focusing on the water content of the fish during fermentation. Generally, the acceptance of fermented fish products varies according to the colour, odour, texture, and flavour. From the study about som-fug, the fish fermented with 10 g of salt and pre-soaked in spices were the most accepted product compared with the ones fermented with 15 g of salt [106]. This is due to the previously discussed ingredient factor in the flavour aspect. Therefore, the sensory attributes of fermented fish, which can be altered and enhanced by the manipulation of the biochemical characteristics, greatly influence the acceptance of the products in the market.

## 7. Analysis of Microbial Characteristics of Fermented Fish

Microorganisms are the vital components of fermenting fish. However, in addition to the unique sensory elements produced by fermented fish, it is also important to control the growth of pathogenic microorganisms to promote the health benefits of fermented fish. Therefore, when studying the process of fermentation or the end product, characterising the microorganisms should be one of the first steps. Generally, the methods can be separated into two categories: molecular methods and culture-based methods [5]. Culture-based methods were applied in various studies. In analysing the microbial community of shidal, the nutrient agar and De Man, Rogosa and Sharpe (MRS) agar were used to enumerate the bacterial colonies and lactic acid bacteria (LAB), respectively [28]. This method showed that LAB and *Bacillus* spp. were predominant the shidal samples. In another study involving a variety of Himalayan fermented fish, the culture-based method was implemented. Different media was used, where LAB were pour-plated with MRS agar, aerobic mesophilic bacteria with plate count agar, and yeasts and moulds with potato dextrose agar and yeast extract-malt extract agar [111]. The 189 strains were successfully isolated from sukako maacha, gnuchi, sidra, and sukuti. Similarly, the microbial analysis of ngari and hentak also implemented the same type of agar for each category of microorganism [7,112]. In addition, total plate count was also determined [7]. Lactic acid bacteria, *Micrococcus*, and *Staphylococcus* were discovered to be dominant in ngari, whereas *Micrococcus* and *Staphylococcus* were dominant in hentak [7]. The examples showed that culture-based methods are commonly used in many studies of fermented fish, and the results paired with biochemical and physiological characterisation are reliable. According to these results, culture-based methods are still relevant to study microbes in fermented fish products. However, more modern methods of microbial analyses are often performed with molecular methods.

Molecular methods such as next-generation sequencing (NGS), polymerase chain reaction (PCR), capillary sequencing, and denaturing gradient gel electrophoresis (DGGE) were implemented in various studies (Table 3). Before sequencing, conventional PCR is usually performed via thermocycler to amplify the genes [26,27,113]. Then, next-generation sequencing technology, the Illumina MiSeq system, was used to process the NGS data of bacterial 16S rRNA from Thai fish sauce samples. The finding showed that *Peptostreptococcus* sp., *Peptoniphilus* sp., *Gallicola* sp., *Fusobacterium* sp., *Halanaerobium* sp., and *Vagococcus* sp. were the most predominant bacterial genera from the samples [113]. The same technology was also adapted in the microbial analysis of yu-lu samples, where the most dominant genus was *Halanaerobium* [26]. The microbial analysis of shidal employed capillary sequencing to sequence the PCR products, where 40 strains of LAB comprising *Lactobacillus plantarum*, *Pediococcus pentosaceus*, *P. acidilactici*, *P. lolii*, *Enterococcus hirae*, *E. lactis*, *E. faecium*, and *E. faecalis* were identified [27]. It is also important to take note that different methods can be employed together to achieve the data, such as using MRS agar fortified with 0.3% CaCO_3_ to differentiate the acid-producing bacteria from other bacteria in shidal, before conducting PCR, followed by sequencing [27]. Furthermore, 16S rRNA sequencing was done to analyse the microbial diversity of surstömming. The findings showed a core microbiota comprising *Alkalibacterium gilvum*, *Carnobacterium*, *Tetragenococcus halophilus*, *Halanaerobium praevalens*, *Clostridiisalibacter*, and *Porphyromonadaceae* [34]. Additionally, multiplex real-time PCR analyses for the detection of botulinic toxin genes in surstömming samples was performed. The genes that encode for botulinic toxins, which include bont/A, bont/B, bont/E, bont/F, and 4gyrB (CP), were absent. Therefore, the risk associated with *Clostridium botulinum* strains is negligible [34]. Next, DGGE is an electrophoretic method capable of detecting differences between DNA fragments of the same size but with different sequences [114]. PCR-DGGE analysis was performed to identify the mycobiota of katsuobushi. This is to ensure the quality assurance of karebushi and traditional moulding technique [6]. According to mould community characteristics, the predominant moulds eventually changed to *Aspergillus chevalieri* and *A. pseudoglaucus* in an about 1:1 ratio [6].

By using these tools to identify the microbial characteristics of fermented fish products, the origin of the raw ingredients and bacterial microbiota in the fermentation tank can be determined, as these factors may affect the product’s quality [113]. It is also important to determine the safety of the fermented fish by identifying pathogenic microorganisms such as *Escherichia coli*, *Coliform*, *Vibrio parahaemolyticus,* and *V. cholerae* [22], as well as histamine-producing bacteria such as *Tetragenococcus halophilus* [115]. Inversely, understanding the microbial community of the fermented fish is the key to identifying its health benefits, especially regarding probiotics [27,34]. Additionally, as observed, fermented fish can have microbiota or mycobiota, or both, depending on the products, as shown in Table 3. The table also demonstrates the tendency of using molecular techniques in recent years compared with earlier publications. Molecular techniques can identify more microorganisms than culture-based methods, as the latter require biochemical analysis and morphological identification of the isolates. This can be attributed to the advancement of sequencing technologies [5]. Thus, microbial analysis is a crucial step in characterising the safety, health benefits, and fermentation parameters of a fermented fish product.

## 8. Safety and Challenges of Fermented Fish, and Its Prevention Measures

The World Health Organization (WHO) states that fermentation is a high-priority technique that calls for particular safety precautions when preparing food for preservation and storing. More than 13 million new-borns and children under the age of five are reported to die each year in tropical areas of the world [116]. Diarrhoea in children under the age of five makes up about 63% of the global burden [117]. The primary cause of diarrhoea is the consumption of foods with inadequate levels of cleanliness. The hygiene standards of a food are contingent on the state of the product’s raw components, preparation, and storage. A food item cooked with water containing harmful microorganisms, for instance, is considered contaminated and thus poses a health risk [116]. There is always a chance that fermented food, especially traditional foods that are naturally fermented, could be contaminated with harmful microorganisms. This is because fermentation makes raw food materials edible without cooking. There have been cases of severe food poisoning caused by the uneven distribution of salt in LAB-fermented fish products and contamination by *Aspergillus flavus* in traditional starter cultures for rice wine and soy sauce. Although most traditional ways of fermenting fish have their own built-in safety features, the final food quality and safety of fermented fish products can still be affected by factors like processing hygiene, histamine poisoning, clostridium poisoning, and salmonella poisoning [118]. Meanwhile, parasitic infections transmitted through food can cause both acute and chronic health issues. It is estimated that 11 major parasite infections cause 48.4 million illnesses annually, of which 48% are transmitted through food [119].

**Figure 5:**
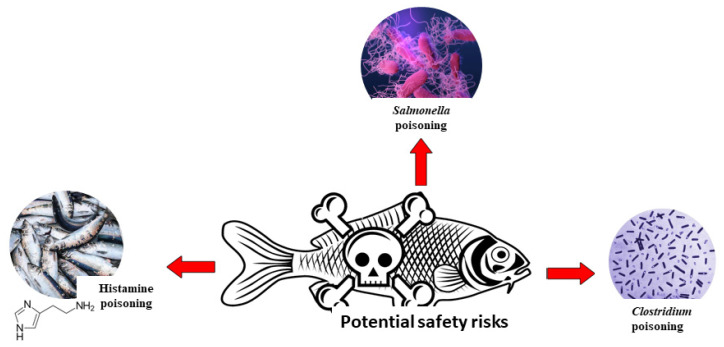
The potential safety risks of fermented fish consumption.

In general, fermented fish products are safe to eat, though a number of factors make the product unsafe, such as the use of contaminated, low grade, or poor quality raw materials; insufficient raw material storage facilities; use of unhygienic preparation techniques; improper handling of products with chemicals; poor hygienic marketing facilities; lack of standard packaging practises; and cross-contamination during marketing that poses risk to purchasers [120]. Due to their relative significance and higher incidence rate, several elements affecting the safety and quality of fermented food items (Figure 5) are highlighted.

### 8.1. Histamine Poisoning

Histamine fish poisoning (HFP) is one of many challenges in food safety (Figure 5) that affects both developed and developing countries around the world [121]. HFP, also called scombroid fish poisoning, is a food-borne illness caused by eating histamine-contaminated fish from the Scombroidae and Scomberesocidae families, such as mackerel, bonito, albacore, and skipjack [122]. This illness is intimately associated with the production of histamine in fish that has been improperly stored after it has been caught [123]. In the majority of instances, histamine levels in disease-causing fish have been above 200 ppm, frequently exceeding 500 ppm [124]. A 42-year-old man came in with a one-hour history of a skin rash that burned and itched, along with a headache and a metallic taste in his mouth resulting from scombroid fish poisoning [123]. The signs of being poisoned by histamine in fish are like those of an allergic response. Between 1998 and 2012, histamine fish poisoning reactions in the US were most often caused by tuna, mahi mahi, escolar, marlin, and salmon [125]. In fact, tuna and mahi mahi alone make up more than 80% of all reported cases [122]. More than 800 persons have developed HFP in the past five years, with 25% of those patients hospitalised in Indonesia [121]. In Kaohsiung City, southern Taiwan, nine people fell ill in April 2017 after eating milkfish surimi products (fish ball), which has been linked to a histamine fish poisoning incident [126].

Basic good manufacturing practices (GMP) coupled with a suitable hazard analysis critical control point (HACCP) system could reduce the potential for histamine poisoning. To meet freshness conditions and prevent the growth of spoilage and histamine-producing bacteria, fish must be stored at a temperature close to that of melting ice as soon as possible after harvest in order to comply with European legislation. Heading, gutting, filleting, chopping, etc. should all be done in a safe and clean manner onboard ships. Furthermore, fresh fishery products must be stored and transported at the specified temperatures so as not to harm food safety [127]. The most important physical and chemical factor affecting amine production was pH. The bacteria are able to adapt to the physical and chemical conditions of the product’s fermentation process, which is a major safety risk [92]. In addition to its toxicity to humans, histamine determination is useful as an indicator of the quality and freshness of fish and fish products. Along these lines, maintaining the safety and quality of fish also depends on consumers taking responsibility for their own fish storage temperatures. Whether in a traditional or modern market, retailers or consumers may have had difficulty maintaining a consistent cold-chain system while purchasing fish for direct consumption at home. As a result, it is important that individuals learn how to handle fish safely, especially Scombroid species. Government and commercial organisations should work together to make sure traditional fish vendors have access to modern conveniences like ice producing machines to succeed. Additionally, the present maximum permissible amount of histamine in fresh fish for direct eating should be reassessed to provide the highest level of consumer safety [121].

### 8.2. Clostridium Poisoning

The bacteria *Clostridium botulinum* produces a toxin that can induce a rare but potentially lethal sickness known as botulism [128]. Botulism, a severe form of food poisoning results when the toxin-containing foods are ingested. *C. botulinum* and its spores can infect foods like fish and products that present a large risk of botulism disease in consumers (Figure 5), making botulism a critical public health issue owing to its high frequency and mortality rate [129]. The four types of botulism that occur in nature include those transmitted through food, found in infants, acquired through wounds, and acquired by colonisation of the adult intestines. Since 2001, 326 cases of foodborne botulism have been reported in the United States, as documented by National Botulism Surveillance conducted by the Centers for Disease Control and Prevention (CDC) [130]. Yearly confirmed cases averaged 19, with a range of 1–63. (range: 2 to 39 cases) [128]. In many cases, *C. botulinum* can thrive in the nutrient-poor environment of fermented fish products [131]. Although this food illness is rare, its mortality rate is high; the 962 recorded botulism outbreaks in the United States from 1899 to 1990 (2) involved 2320 cases and 1036 deaths [132]. In Japan, Izushi (traditional fermented fish preserved in rice), is produced by allowing fish or roe to ferment in a jar of steamed rice. One such case is the deadly outbreak of 1951 in Iwanai Hokkaido, where izushi-borne type E botulism was suspected. The cause of the outbreak was traced back to *C. botulinum* type E (Iwanai) [133]. Meanwhile, an outbreak of botulism type E was discovered in Egypt after people ate feseekh, a fermented and salt-cured fish dish traditionally made with uneviscerated grey mullet [134]. Consumers can take several steps to protect their family from botulism at home: Low-acidity meals canned at home should be heated to 176 °F (80 °C) for at least 30 min before consumption. Twenty minutes of heating time is recommended for canned goods including maize, spinach, and meats [135].

### 8.3. Salmonella Poisoning

In Asia, it is common practice to ferment raw fish to increase its shelf life and safety; nevertheless, the persistence of harmful bacteria in the fermented product is less understood. *Salmonella* and other *Vibrio* species (Figure 5) are among the bacteria that can contaminate raw seafood [136]. *Salmonella enterica* is a varied bacterium with over 2500 potentially pathogenic serotypes *S. enterica* contamination of seafood is not only dangerous to consumers’ health, but also costly when imports are rejected [137]. *Salmonella* contamination was discovered to be widespread in the three fish landing facilities and seven retail fish markets of Mumbai, India [137]. Garlic is one of the often-used spices in fermented fish, and because it has an antibacterial action against pathogenic bacteria commonly found in fish products, it can contribute to the product’s safety without affecting the fermentation. Combining garlic with garlic-fermenting starting cultures could increase the safety of fermented fish products to prevent contamination of *Salmonella* [136].

### 8.4. Hygiene of Processing Conditions

Hygiene refers to the conditions and steps that must be taken during processing to make sure that the products are safe and fit for human consumption. Time, temperature, and cleanliness are all important parts of the process of preparing fish and fishery product. Speedy work, good icing, and clean processing operations go a long way towards reducing spoilage [138]. It is important to store products at the right temperature after you get them if you want to keep them safe and in good shape. Chilled products should be stored in a clean way at temperatures less than or equal to 4 °C, products in modified atmosphere packaging (where the air around the fish is different from normal air) at 3 °C or lower, and frozen products at temperatures less than or equal to −18 °C [139,140]. It is critical to implement sanitary and hygienic standards during fish processing to reduce loss caused mostly by microorganisms, which are pervasive in our environment. Hygiene practices and facilities should be designed in such a way that an appropriate level of personal hygiene can be sustained to prevent contamination. Facilities and equipment must include suitable methods to wash and dry hands, as well as adequate restroom and changing facilities for staff that are appropriately placed and designated. All employees in a facility should maintain a high level of personal cleanliness and take all necessary precautions and they should be aware of their role and responsibilities in preventing contamination and deterioration of fish. Besides, the handlers must also have the essential knowledge and skills to handle fish safely [139,140].

Equipment and utensils should be made of materials that are strong, non-toxic, non-absorbent, resistant to corrosion, will not rust, and are easy to clean and disinfect. Bamboo and wood utensils are not allowed. The business must have a separate quality control department with qualified staff that matches its processing capacity. It must also have the necessary protocols, standards, facilities, and equipment for sample testing. The equipment must be checked and calibrated according to the standards that apply. The business should be able to do simple tests like water quality tests and microbial tests. Tests and inspections must be recorded [141]. Major obstacles that impacted fish post-harvest operations included a lack of cold storage facilities and unpredictable weather [70]. Accordingly, it is suggested that new technology, self-development skills for fishing communities, and improved access to freezer facilities be implemented to reduce the financial loss caused by post-harvest fish losses [70].

## 9. Advanced Technology in the Progress of Fermented Fish and Fish Products

An excellent example of a hurdle technology is fermentation. It functions as a preservation strategy by reducing the substrate’s pH, redox potential (Eh), and a_w_. By adding lactic bacteria (LAB) to the fish to be fermented, fermentation is occasionally referred to as bio-preservation in modern cooking techniques. LAB-produced antimicrobials include lactic acid, acetic acid, antimicrobial nisin, hydrogen peroxide, and peptide bacteriocins. These active ingredients stop the growth of pathogenic and spoilage microorganisms, aiding in the preservation of the fish [120,142]. Traditional methods for producing fish products include limited drying, smoking, fermentation, and using substrates in a way that encourages the growth of some beneficial microorganisms that stop the growth of pathogenic microorganisms [92]. This technology offers benefits for upcoming applications because it is safe, environmentally friendly, and low energy consuming [143]. In Japan, filamentous fungus like KOJI is commonly used to make fermented fish items because of the positive effects they have on the quality of the final product. Fermented cereal KOJI is commonly used as a starter culture for making miso, soy sauce, sake, and other fermented foods and drinks. Key enzymes like amylase, protease, lipase, and others are produced by the KOJI fungus. Some examples of KOJI are bacteria and yeasts; however, they are not the only ones. The use of mixed cultures in the fermentation of filamentous fungi results in increased nutritious and functional value in the fermented food, as well as greater efficiency in the material conversion process [144].

In recent years, the use of modern fermentation techniques in meat and fish has piqued the interest of an increasing number of researchers. Food safety has been improved, processing time has been reduced, and product sensory properties have been altered by inoculating starter cultures into meat and fish [145]. Fish products are common foods with high levels of biogenic amines (Bas) and excessive consumption may pose health risks. As a result, it is critical to demonstrate the mechanism of formation and control of Bas during the fermentation of fish sauce. A novel method for controlling Bas in fermented foods was discovered through the contribution of microorganisms that control the accumulation of Bas in fish sauce fermentation. *Staphylococcus nepalensis* 5-5 and *Staphylococcus xylosus* JCM 2418 were found to be potential starters for BA control [146]. A comparative UHPLC-Q/TOF-MS-based metabolomics approach combining equivalent-quantification was used to evaluate the taste qualities and characterise metabolite profiles in fish product such as tilapia fillets and Chinese fish sauce during fermentation. This approach identified chemical components and sheds new light on the taste, nutrition, texture, and flavour quality of fish products [43,147].

On the other hand, nutritional composition can be uncovered at the molecular level using foodomics. Since the term “foodomics” was introduced a decade ago, these omics technologies have received a lot of interest in contemporary food, nutrition, and health research [148]. Analytical approaches from many omics disciplines, such as proteomics, metabolomics, lipidomics, nutrigenomics, metagenomics, and transcriptomics, have recently fuelled food-related research. The use of various omics technologies, either individually or in combination, to analyse food ingredients as well as to authenticate food and assess food safety and quality is the subject of numerous studies [149]. Analyses of food pathogens and tainted food using next-generation sequencing technologies provide valuable insight into the infectious process, disease outbreak, and post-treatment microbial activity [150]. An investigation was conducted through DNA-sequencing analysis to examine the microbiota found in fermented fish items in several villages in northeastern Thailand (pla-ra). The microbial communities in pla-ra provide a better knowledge of how traditional products are formed. Advanced fermentation process optimization, such as utilising dominant bacterial taxa in starter cultures, may enhance the fermentation of food, manage food quality, and provide helpful guidance for industrial applications [151]. Nowadays, the fish waste disposal is a significant environmental issue. Additionally, the fermentation of fish waste yields other noteworthy substances. These substances can be utilised to produce compounds that are useful in the development of nutraceuticals [143]. The fish waste is a good source of bioactive substance since it contains a significant amount of high-quality protein. The fermentative bioprocess converts fish waste into usable functional chemicals while also reducing the environmental impact of production; thus, it can be considered a “clean production” [152].

In conclusion, a variety of fermented fish products are produced in different regions of the world. Even so, the physiochemical and biochemical changes that occur to various fermented fish were similar, forming trends for each parameter studied. Furthermore, the safety challenges of fermented fish mainly covered different types of poisoning and hygiene processes. The processing and safety assessment of fermented fish products lies in the advancement of processing technology and other advanced approaches for quality assessment.

## 10. Future Perspectives

Although fermented fish products are commonly consumed in certain regions of the world, this regional delicacy is yet to be widely commercialised worldwide. However, the globalisation of local fermented fish products is expected to occur in the near future, due to a deeper understanding of the fermentation process. Additionally, the health benefits associated with the consumption of fermented fish could expand the market of fermented fish products. The future direction in fermented fish products involves improving the fermentation process via alterations and enhancements of fermentation parameters. As previously mentioned, the degradation of lipids and proteins are responsible for both positive and negative attributes of fermented fish, whereby desired substrates are yielded as well as harmful ones like histamine. To maximise the potential of fish fermentation, the temperature, pH, and fermentation time need to be optimised according to the types of fish used in accordance with the desired type of fermented fish product. Additionally, more in-depth studies on the proximate compositions of fermented fish should also be conducted to develop stronger foundations and directions on how to improve its sensory attributes. Systematic measures, such as Basic Good Manufacturing and Hygiene Practices and implementation of the Hazard Analysis Critical Control Point (HACCP) system could significantly alleviate the issues with fermented fish safety. Future perspectives on fermented fish products also lie in the advancement of technology for the fermentation process and quality assessment. Most studies cited in this review focused on the characterisation of fermented fish in terms of biochemical and microbial properties and its safety. The utilisation of omics studies in assessing the microbial community of fermented fish products is expected to be more prominent. The advancement of technology in fermented fish processing is also an important direction, where the goals are to enhance the processes and address the issue of sustainability.

## Figures and Tables

**Table 2:** Fermented fish products and their respective proximate composition.

Fermented Fish Products	Moisture (%)	Lipids (%)	Protein (%)	Ash (%)	Minerals (mg/g)	References
Momone (*Cassava croaker*)	57.6	2.4	26.2	14.6	-	[70]
Momone (Grey snapper)	64.9	1.4	18.2	-	-	
Jambal roti (giant sea catfish)	48.53	0.21	5.49	18.02	-	[71]
Hentak (Indian flying barb)	36.30	13.60	33.38	11.43	-	[72]
Ngari (*Puntius sophore*)	36.03	13.34	38.38	5.49	-	
Shidol (*Puntius sophore*)	33.44	20.31	38.35	7.19	-	[73]
Shidol (*Setipinna phasa*)	37.52	24.1	27.2	10.2	-	
Adjuevan (*Chloroscombrus chrysurus*)	69.65–71.25	12.16–12.36	21.21–26.81	10.5	4.16 (Ca) 3.82 (P), 0.72 (Na), 0.67 (K).	[74]

**Table 3:** Microbial diversity of various fermented fish products.

Product	Techniques/Methods	Microbial Community	Reference
Shidal	MRS agar to differentiate the acid-producing bacteria from other bacteria Conventional PCR Capillary sequencing	LAB *Bacillus* spp.	[27]
	Total Plate Count (TPC) MRS agar for LAB	*L. plantarum* *Pediacoccus pentosaceus* *P. acidilactici* *P. lolii* *E. hirae* *E. lactis* *E. faecium* *E. faecalis*	[28]
Himalayan fermented fish (sukako maacha, gnuchi, sidra, and sukuti)	Plating for enumeration and cell morphology MRS agar for LAB Plate count agar for aerobic mesophilic bacteria Potato dextrose agar and yeast extract-malt extract agar for yeasts and moulds	*Lactococcus lactis* subsp. *cremoris* *L. lactis* subsp. *lactis* *L. plantarum* *Leuconostoc mesenteroides* *Enterococcus faecium* *E. faecalis* *Pediococcus pentosaceus* *Weissella confusa*	[111]
Ngari and hentak	Plating for enumeration and cell morphology MRS agar for LAB Plate count agar for aerobic mesophilic bacteria Potato dextrose agar and yeast extract-malt extract agar for yeasts and moulds	Ngari: LAB *Micrococcus* *Staphylococcus* Hentak: *Micrococcus* *Staphylococcus*	[7]
		*Lactococcus lactis* subsp. *cremoris* *Lactococcus plantarum* *Enterococcus faecium* *Lactobacillus fructosus* *Lactobacillus amylophilus* *Lactobacillus coryniformis* subsp. *torquens* *Lactobacillus plantarum* *Bacillus subtilis* *B. pumilus* *Micrococcus* *Candida* *Saccharomycopsis*	[112]
Thai fish sauce	Conventional PCR 16S rRNA sequencing via Illumina MiSeq Reads analysis via bioinformatics	*Peptostreptococcus* sp. *Peptoniphilus* sp. *Gallicola* sp. *Fusobacterium* sp. *Halanaerobium* sp. *Vagococcus* sp.	[113]
Yu-lu	Conventional PCR Genome sequencing via Illumina MiSeq Reads analysis via bioinformatics	*Halanaerobium*	[113]
Surstömming	Conventional PCR 16S rRNA sequencing via Illumina MiSeq Reads analysis via bioinformatics	*Alkalibacterium gilvum* *Carnobacterium* *Tetragenococcus halophilus* *Halanaerobium praevalens* *Clostridiisalibacter* *Porphyromonadaceae*	[34]
Katsuobushi	Conventional PCR DGGE analysis	*Aspergillus chevalieri* *A. pseudoglaucus*	[6]

## Data Availability

No new data were created or analyzed in this study. Data sharing is not applicable to this article.
